# Genome-Wide Comparisons of Phylogenetic Similarities between Partial Genomic Regions and the Full-Length Genome in Hepatitis E Virus Genotyping

**DOI:** 10.1371/journal.pone.0115785

**Published:** 2014-12-26

**Authors:** Shuai Wang, Wei Wei, Xuenong Luo, Xuepeng Cai

**Affiliations:** State Key Laboratory of Veterinary Etiological Biology, Lanzhou Veterinary Research Institute, Chinese Academy of Agricultural Sciences, Lanzhou, Gansu, China; University of Cincinnati College of Medicine, United States of America

## Abstract

Besides the complete genome, different partial genomic sequences of Hepatitis E virus (HEV) have been used in genotyping studies, making it difficult to compare the results based on them. No commonly agreed partial region for HEV genotyping has been determined. In this study, we used a statistical method to evaluate the phylogenetic performance of each partial genomic sequence from a genome wide, by comparisons of evolutionary distances between genomic regions and the full-length genomes of 101 HEV isolates to identify short genomic regions that can reproduce HEV genotype assignments based on full-length genomes. Several genomic regions, especially one genomic region at the 3′-terminal of the papain-like cysteine protease domain, were detected to have relatively high phylogenetic correlations with the full-length genome. Phylogenetic analyses confirmed the identical performances between these regions and the full-length genome in genotyping, in which the HEV isolates involved could be divided into reasonable genotypes. This analysis may be of value in developing a partial sequence-based consensus classification of HEV species.

## Introduction

Hepatitis E virus (HEV) is an important public health concern in developing countries [1]. It is a non-enveloped, positive-sense, single-stranded RNA virus that is fecal-orally transmitted through contaminated food and water. It has an approximately 7.2 kb genome consisting of a short 5′-untranslatedregion (UTR), three partially overlapping open reading frames (ORFs1–3), and a 3′-UTR terminated by a poly(A) tract [2].

Although only one serotype has been determined until now, HEV displays extensive genetic diversity [3]. Generally, based on the variability of the full-length genome sequence among different strains, HEV has been classified into four major genotypes that infect humans (HEV-1, HEV-2, HEV-3 and HEV-4) and several additional genotypes like HEV-5 and HEV-6 [1], [3]–[5]. The later genotypes have been variously assigned to some HEV strains isolated from species like avian and rats [6]. As isolates closely related to genotypes 1–4, sequences from wild boars (WB) have been considered as additional genotypes (namely genotype 5 and genotype 6 as well) [7] and sequences from rabbits (Rab) appear to be sub-genotypes from genotype 3 [6]. More recently, the genotype HEV-7 was proposed to for HEV isolates from camels [6].

In many previous studies, sequence comparisons and phylogenetic analyses were performed to determine the genotypic distribution of HEV isolates, extensively based on partial genomic sequences [8]–[11]. Consequently, different studies have assigned HEV genotypes based on multiple genomic regions and no commonly agreed standard classification scheme based on partial genomic regions has been proposed. The underlying genotyping results are reasonably related to the genomic regions analyzed and the length of sequences examined, since different research groups have analyzed different regions of different sizes [4], [5], [12]. Partly because of the inconsistence of different classification criteria, subsequent studies have differed in their assignment of some HEV isolates to genotypes [1], [3], [13]. The sequencing of smaller regions of the genome is simpler and less time-consuming than whole genome sequencing, and data for various sequence subsets may be more available than whole-genome sequences for some isolates. However, the use of different genomic regions with different sizes for HEV genotyping by different research groups makes it difficult to compare those results, complicates the use of any classification and sometimes yields inconsistency in genotyping [1], [3], [12], [13].

Statistical and phylogenetic methods have been used in several studies [3], [13]–[16] to investigate the feasibility of using some partial genomic regions to reproduce phylogenetic referencing based on full-length genomes. And several genomic regions showing good phylogenetic correlations with the full-length genomes in HEV genotyping have been found [3], [13]–[16]. Particularly, in the study by Zhai *et al*. [3], the authors have performed a statistical comparison of the phylogenetic performance of several individual genomic regions having been used for HEV genotyping and found the MJ-C region in the viral RdRp domain could provide sufficient information to replace the full-length genome for HEV genotyping. Another study based on a score method only involved five genomic fragments in their analysis [16]. However, the genomic regions examined in these studies were limited, which may not completely reflect the useful phylogenetic signal for every genomic region in HEV genome. No study has investigated the phylogenetic performances of different genomic regions from a genome wide to identify a consensus genomic region that could provide reliable genotype referencing. Moreover, the situation has been further compounded by the recent observation that synonymous substitutions may be saturated in comparisons between and within HEV genotypes [13], which could confuse classification results. Thus, a genome-wide investigation of phylogenetic performance that takes the problem of sequence saturation into consideration for HEV genome is urgently needed.

In this study, we applied a strategy of calculation of genetic distance matrix correlations between genomic regions and the full-length genome to compare phylogenetic performance between them and estimate the feasibility of using the corresponding individual genomic regions to represent the complete genome in genotyping [17]. This approach, with a methodology similar to the mirror-tree strategy [18]–[20], is based on calculating the correlation coefficient (r-value) between genetic distance matrices of various genomic regions and that of the complete genome and thus can evaluate the phylogenetic performance of each partial sequence without tree constructions. Using a sliding window analysis, every partial sequence in HEV genome was evaluated, taking sequence saturation into account. This study may be of value in developing a partial region-based consensus classification for HEV genotyping.

## Materials and Methods

### Data selection

Complete genome sequences were obtained from recent phylogenetic analysis studies [11], [13] and were downloaded from Genebank on February 10, 2014. Sequences have been removed if there was evidence of recombination, if they differed from any other sequence of the dataset by <2% of nucleotide sites (excluding the hypervariable regions [HVR]) and if too many ambiguous gaps (>5%) during the alignment were generated when they are added. Totally, 101 sequences were used: DQ459342.1, AB248522.1, EU366959.1, EU360977.1, EU375463.1, AY723745.1, EF570133.1, M74506.1, M73218.1, AB369687.1, M94177.1, X98292.1, X99441.1, AF060669.1, AF076239.3, AJ272108.1,AP003430.1, AF459438.1, AB074915.3, AB089824.1, AB193176.1, AB197674.1, AB222182.1, AB197673.1, AB222183.1, AB246676.1, AB220974.1, AB253420.1, AB222184.1, AB236320.1, EF077630.1, AB248520.1, AB290312.1, AB290313.1, M80581.1, AB074920.3, AB091394.1, AF455784.1, AB369689.1, AB369691.1, AB369690.1, AB291963.1, AB291967.1, FJ763142.1, JQ013791.1, EU495148.1, FJ457024.1, AB630970.1, AB291953.1, AB630971.1, JF915746.1, JQ013792.1, JQ013795.1, JQ013793.1, AB073912.1, AY115488.1, AY594199.1, DQ279091.2, EU723512.2, EU723514.2, FJ610232.1, EU723513.1, EU723516.1, AB481227.1, AB481228.1, AB481229.1, FJ998008.1, EU676172.2, GU119960.2, GU119961.3,AB291960.1, FJ426403.1, AB481226.1, GU188851.1, FJ426404.1, AB291961.1, GU937805.1, HM152568.1, HM439284.1, GU361892.1, AB291962.1, FJ527832.2, FJ705359.1, FJ653660.1, FJ906895.1, HQ634346.1, AB591733.1, AB591734.1, FJ906896.1, FJ956757.1, AB108537.1, AY204877.1, AY575857.1, GU206559.1, AB189070.1, AB369688.1, AB602441.1, AB573435.2, AB602440.1, AY230202.1, AB161719.1.

### Data processing and methodology of sliding window analysis

The ORF and protein sequences for each genome were determined using the genome annotation in GeneBank and verified manually. Three data sets constructed based on all the sequences were used as reference sequences for further analysis: data set 1, comprising nucleotide sequences of the completed genomes; data set 2, comprising of nucleotide sequences of concatenated ORF1/ORF2 sequences with all the third codon positions removed; and data set 3, comprising of amino acid sequences of the concatenated ORF1/ORF2 coding regions.

For each reference data set, overlapping windows with different sizes shifted by one site were sliding over them, generating continuous partial sequences of the corresponding sliding windows sizes. For reference data set 1, window sizes of 300,400 and 500 nucleotides were applied to the analysis. For reference data set 2, window sizes of 300 and 400 nucleotides were used. And for reference data set 3, the window size was set to 200 amino acids. If not specially defined, all the positions mentioned in this study were labeled according to the strain SAR55 (GeneBank ID: M80581). The genome region at the start of ORF1 which is missing in this strain was not used in our analysis.

### Calculation of correlations between genetic distance matrices

The extent of agreement between the phylogenetic similarities of two sequences was assessed using a strategy similar to the mirror-tree approach [17], [19], [21] by comparing their distance matrices. The extent of agreement between two genetic matrices is evaluated using Pearson's correlation coefficient (r-values). As this method is directly based on the genetic distance matrix between the two sequence alignments, the resulting r-values can give estimations of phylogenetic relationship in distance-based phylogenetic analyses [18], [19].

As an extension of this method, a strategy of sliding-window was used to evaluate the phylogenetic correlations between different partial sequences and the full-length reference sequences from a genome-wide scale. For each sliding window, the correlation between the sequence within it and the corresponding data set was calculated as follows:

All data sets were aligned by using the program mafft [22] (–auto) with reference to the protein sequence alignment. Pair-wise distance matrices of each sliding window sequence alignment and of the reference data set alignment were constructed by Mega-CC [23] using p-distance model with gaps completely deleted. For a partial sequence X within a sliding window and the full-length reference data set (data set 1 or concatenated data sets) Y, the correlation (r-value) between genetic distance matrices was calculated according to the following equation [24]:
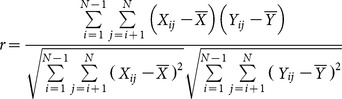
where X_ij_ represents the genetic distance from HEV isolate i to isolate j in the distance matrix of sliding-window X; 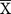 is the mean of all X_ij_ values; Y_ij_ represents the distance of isolate i to isolate j in the distance matrix of reference data set Y; 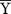 is the mean of all Y_ij_ values; and N represents the number of HEV isolates in the matrices.

All the HEV isolates collected were used in the analyses. For comparison, phylogenetic trees based on the p-distance obtained were constructed using Mega 5.0 [25] (Neighbor-joining method, with other parameters default).

## Results

### R-values between partial nucleotide sequences and the complete genome

**Figure 1:**
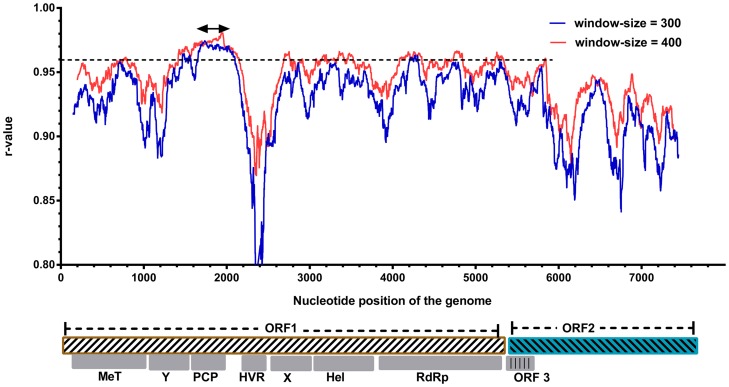
Sliding-window analysis of correlations between genomic regions and the full-length genome (data set 1). The r-values were calculated for overlapping windows of 300 nucleotides (blue) and 400 nucleotides (red) shifted by one nucleotide and plotted against the midpoint of the window. The nucleotide positions represent the site positions in the alignment. The positions of the three open reading frames are shown along with the approximate positions within ORF1 of the methyl transferase (MeT), Ydomain (Y), papain-like cysteine protease (PCP), hypervariable region (HVR), X domain (X), helicase (Hel), and RNA-dependent RNA polymerase (RdRp). The dashed line indicates the r-value  = 0.96, for better comparison.

Compared with the complete genome sequence, the evolutionary rates of the HEV isolates can markedly vary among the different genomic regions (Fig. 1 and S1 Fig.). Although the r-values are slightly variable with different sizes, similar evaluations can be obtained for a given genomic region. For the 300 nucleotide window, several r-value peaks above 0.96 within different genomic regions (above the dotted line) could be found throughout the whole genome. The highest peak was located in the papain-like cysteine protease domain (PCP) of ORF1, with an r-value of 0.9747 (1548–1848, reference M80581), whereas the lowest value was located in the hyper-variable region (HVR). And a extensive region flanking the highest peak, before the HVR, could show very high correlations with the entire HEV genome (positions 1464–2100), where any continuous sequence region of 300 or more nucleotides could exert a relatively higher r-value with the whole genome. Another genomic region that has r-values greater than 0.96 is located within the RNA-dependent RNA polymerase (RdRp) domain (3738–4109). Though several peaks were found in ORF2, no regions having r-values greater than those of the PCP domain could be detected. Similar findings were obtained for all the window-sizes examined.

### R-values based on the concatenated first two codon positions and amino acid sites

**Figure 2:**
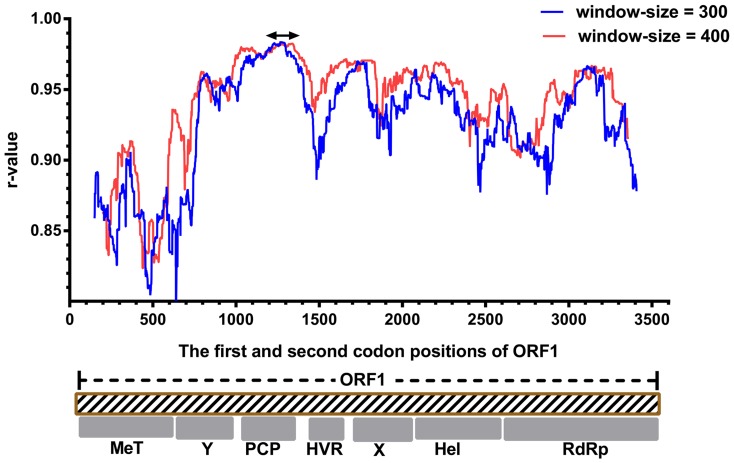
Sliding-window analysis of correlations of the first and second codon positions in ORF1/ORF2 (data set 2). The window sizes and plotting conditions are the same as in Fig. 1. The nucleotide positions represent the site positions in the alignment of ORF1. The arrow indicates the highest r-value.

**Figure 3:**
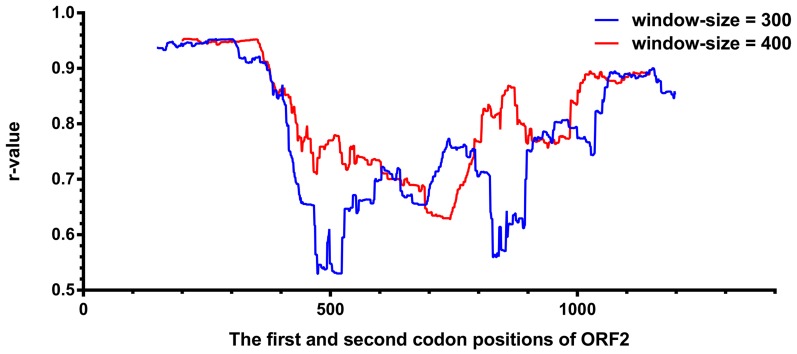
Sliding-window analysis of correlations between the first two codon sites in ORF2 and those in ORF1/ORF2 (data set 2). The window sizes and plotting conditions are the same as in Fig. 1. The positions represent the site positions in the alignment of ORF2.

For nucleotide sequences of the coding regions, all third codon positions were excluded from the analyses due to potential mutational saturation. The remaining data were partitioned by codon positions. As observed for the concatenated first two sites in ORF1 (Fig. 2), the r-values calculated from reference data set 2 varied more dramatically than those in the analysis of reference data set 1. The r-values peaked (r-value  = 0.9837) in the PCP domain of ORF1 at a window size of 300 nucleotide sites (1672–2122 in ORF1). The genomic regions (1596–2169 in ORF1) flanking this peak also showed relatively high r-values (>0.98). There were also several regions of very low r-values, including one near the beginning of ORF1, one located in the HVR and one located in the helicase (Hel) and RdRp domains (approximately 3210–3840 in ORF1). For ORF2 (Fig. 3), only the sequence located at the 5′-region, approximately corresponding to the region where ORF2 and ORF3 are encoded by overlapping reading frames could show high r-values, among which the highest r-value is 0.953 (a window-size of 300).

**Figure 4:**
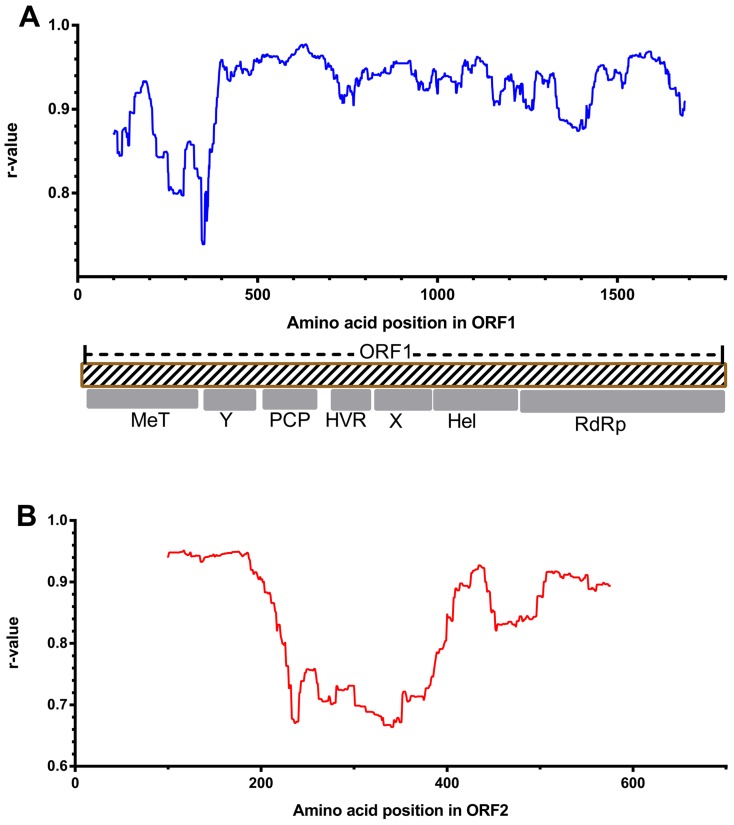
Sliding-window analysis of correlations between partial protein sequences and the complete concatenated protein sequences of ORF1/ORF2 (data set 3). The r-values were calculated for overlapping windows of 200 amino acids in ORF1 (A) and ORF2 (B) shifted by one amino acid and plotted against the midpoint of the window. The x-axis represents the positions in the amino acid alignments of ORF1 (A) and ORF2 (B).

When sequences approached saturation among some virus isolates, amino acid sequences may be an alternative way to provide efficient phylogenetic estimation. We therefore investigated the r-values between partial amino acid sequences in each protein (coding by ORF1 and ORF2) and the full-length amino acid sequences of concatenated ORF1/ORF2 respectively (reference data set 3). Sliding-window analysis revealed that for ORF1 (Fig. 4), r-values peaked at the region of positions 510–710 in PCP domain (window-size  = 200), similar to the peak region determined by nucleotide analysis. However the region near the beginning of ORF1 had relatively low r-values with the reference data set 3, while the region at the end of ORF1 also showed high r-values. For ORF2, reference data set 3 analysis found results similar to the reference data set 2, with high r-values at the beginning of ORF2.

### Phylogenetic analysis

**Figure 5:**
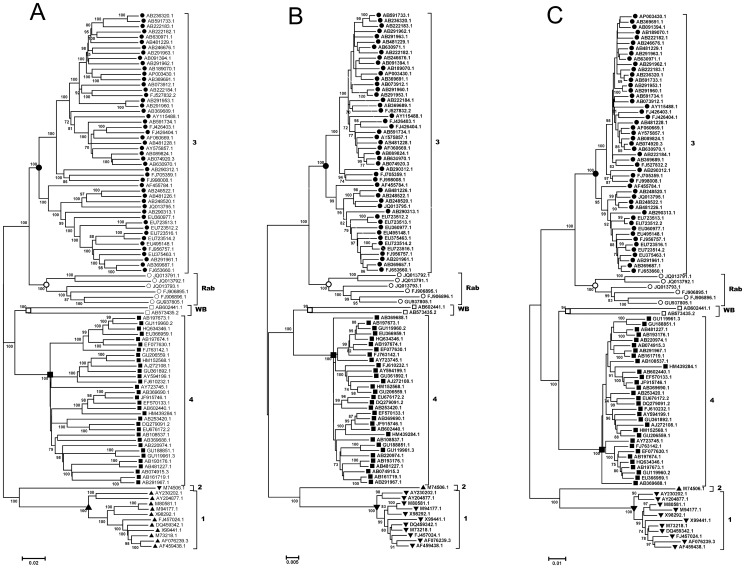
Phylogenetic analysis of HEV isolates based on the three reference data sets. Neighbor-joining trees were produced by using nucleotides of complete genome (data set 1) (A), using of nucleotides concatenated first and second codon sites of ORF1/ORF2 (data set2) (B), using amino acids of concatenated protein sequences of ORF1/ORF2 (data set 3) (C). Bootstraps values (>70) and scale bars are shown.

**Figure 6:**
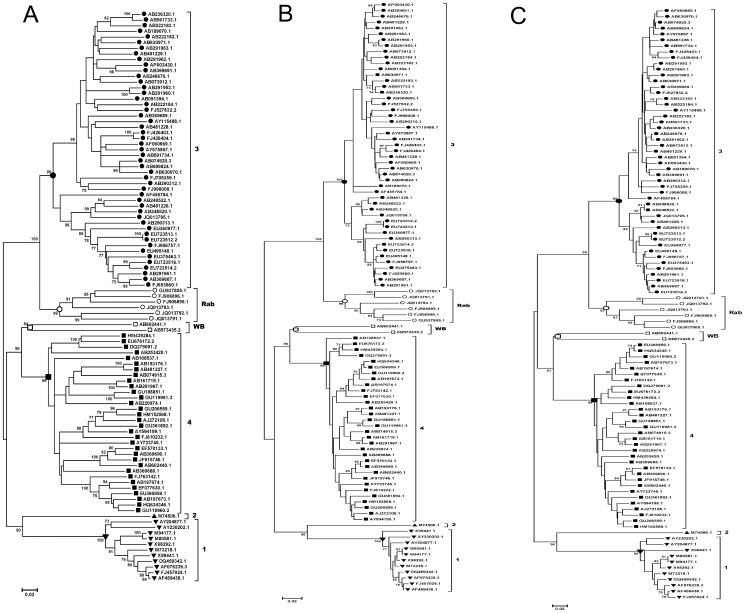
Phylogenetic analysis of HEV isolates based on ORF1 regions with the highest r-values. Neighbor-joining trees were produced by using nucleotides of the genomic region at PCP domain with a length of 400 nucleotides (1737–2137) (A), 400 first two codon sites (1052–1452) (B), and the amino acid sequences (510–710) (C). Bootstraps values (>70) and scale bars are shown.

In order to confirm the phylogenetic performances of the short genomic regions or coding sequences which have the highest r-values in the above analyses, phylogenetic trees were constructed based on the genomic region at PCP domain with 400 nucleotide sites in ORF1 (1737–2137), with 400 first two codon sites in ORF1 (1052–1452), and with the amino acid sites in ORF1 (510–710) respectively. And as a comparison, the three large-size data sets were also used for tree constructions. NJ trees based on p-distances of these data sets with a 1000 bootstrap replications were obtained. All the trees based on the reference data sets showed very similar topologies, all of which classify these HEV stains into the four known human-related genotypes and two additional groups for WB isolates and Rab isolates (Fig. 5). The branches leading to genotype groups were supported by nearly 100% of bootstrap replications in all cases. When the three partial sequences in ORF1 were used for phylogenetic analyses, similar tree topologies and genotype groups with reliable bootstraps supported were also observed, with all the four known genotype virus strains, as well as the variants from Rab and WB, clearly clustered (Fig. 6). During our analysis, some phylogenetic trees based on other genomic regions were also constructed. We found significant genotype inconsistency between trees based on regions of high r-values and those based on regions of low values. For instance, the tree constructed based on a region in ORF2 (5605–6004, r-value  = 0.87) showed a dramatically different topology to the complete genome tree, and failed to clearly classify the known genotypes, particularly for the rabbit strains and WB strains (S2 Fig.).

## Discussion

Partial genomic sequences instead of the complete genome have been successfully applied to genotype referencing for hepatitis B virus [26] and hepatitis C virus [27]. However, for HEV, no commonly agreed criteria of genotyping have been proposed. Besides complete genome sequences, a variety of sub-genomic regions have been used to make taxonomic assignments, which has complicated and obscured the classification of HEV genotypes [1], [3], [4], [7], [13], [28], [29]. This study tried to find and demonstrate short consensus genomic regions from a genome scale that can be alternatively used to reproduce HEV genotype assignments based on the full-length genome.

By using a genome-wide sliding window analysis, we made comparisons of phylogenetic correlations (r-values) between all possible partial genomic regions and the full genome. The r-values are calculated based on the genetic distance matrices and will give an estimation of genotypic relatedness between sequence data sets [17]–[19], [21]. We found the r-values between partial genomic regions and the complete genome varied over the genome. This observation reasonably suggested that phylogenetic analyses based on different genomic regions may give inconsistent phylogenies in some cases and, thus, produce inconsistency in HEV isolate genotyping, which has been observed by some previous studies [3], [15], [16], [30] as well as this study (S2 Fig.) when different regions were used Based on analyses of different window sizes, we found several genomic regions showed very high r-values with the complete genome, mostly involving regions in ORF1. Among them, the region located at the 3-terminal of PCP domain (corresponding to positions 1548–1848), before the HVR, showed the highest r-value in our analysis. Our phylogenetic analysis based on this region of 400 nucleotides demonstrated its highly similar performance in genotyping to that of complete genome sequence for the HEV isolates examined (Fig. 5 and Fig. 6), from which all the known genotypes and two additional groups of HEV variants derived from rabbits [28] and wild boars [7] could be reasonably determined.

These results suggest that the regions having high r-values with the full-length genome may have the priority to serve as candidates for representing the whole genome in genotyping of HEV. In the study by Zhai et al. [3], the genomic region MJ-C (4254–4560 in M73218) has been suggested as the best genomic region for genotyping. In our study, we also found it is located in a region with very high r-values (larger than 0.965, window-size  = 400 nucleotides) in the RaRd domain. However, this region did not have the highest r-value in our analysis by a genome-wide screening. In contrast, there were some regions that failed to provide enough or reliable phylogenetic signal to reproduce genotyping results based on a full-length genome analysis. For instance, the HVR had very low r-values and have not been recommended to serve as a maker region for phylogenetic analyses [13]. Additionally, the difficulty in aligning this region also makes it not suitable to be used in phylogenetic analysis. However, this does not mean that the HVR region is not useful for HEV genotyping. Because of the distinctive genotype-specific sequence motifs in this region, it has unique advantages in genotyping HEV isolates just by examining the sequence signatures specific to HEV genotypes rather than traditional phylogenetic analysis [31].

Phylogenetic relationships can be obscured by the analyses based on sequences which involve extensive substitutions that have become saturated. For HEV, comparisons between the HEV genotypes infecting humans have been reported to approach saturation at synonymous sites throughout the genome [13]. Thus, the genotype classification of HEV isolates and its variants may sometimes be more accurate when these mutation saturation sites are excluded from phylogenetic analyses. In our analysis, we found phylogenetic trees produced by using distances at the first and second codon sites or amino acid sites of concatenated ORF1/ORF2 were very similar to the tree constructed based on the complete genome sequence, with no inconsistency in genotyping and sub-typing. This implies that sequence saturation at third codon position may have a limited impact on the phylogenetic analysis of these HVE isolates. In ORF1, using the specific sequence lengths in this study, a region overlapping with the best region in the analysis of reference data set 1 had the highest r-value in reference data set 2, and nearly corresponded to the best region in the analysis in reference data set 3. Moreover, all the trees constructed based on the regions having highest r-values in the respective analyses of the reference data set 2 and the reference data set 3 gave reasonable inferences for HEV sequence relationships (Fig. 6). Thus, in our analysis, the region in the PCP domains can reproduce genotypic taxonomic assignments determined by the complete genome sequence, even when sequence saturation is taken into consideration. However, according to the results based on reference data sets 2 and 3, some partial sequence sites from the concatenated first two codon site sequences or amino acid sequences may not provide enough and reliable phylogenetic signal for genotyping, especially in some regions in ORF2. In ORF2, some regions (Fig. 1, Fig. 2, and Fig. 3) showed relatively low r-values with their reference data sets, and may give unreliable genotyping results inconsistent with those of their reference data sets, especially when the amino acid sequences or the first two codon sites were used. It is reasonable that some regions, such as parts of MeT domain and Y domain, are evolutionarily highly conserved, leading to dramatically reduced divergence at non-synonymous sites and less phylogenetic signal for grouping [13]. This phenomenon suggests that some highly conserved regions of HEV provide only very limited phylogenetic signal, due to infrequent non-synonymous site mutations, and therefore may not be suitable for phylogenetic analyses.

Nevertheless, the method in present study based on calculation of correlations between genetic matrices of partial genomic regions and genome references may be inappropriate for classifying HEV subtypes, as some studies of complete genome sequences have reported that it is not possible to define discrete boundaries that distinguish sub-genotypes with consistency [1], [6], [13], [15].

## Conclusions

Collectively, when considering different levels of phylogenetic analyses (nucleotides, concatenated first two codon sites and amino acids), the genomic region in the PCP domain of ORF1, (1548–1848 in the genome nucleotide sequence, 1114–1414 in the concatenated first two codon sites of ORF1, and 510–710 in the protein sequence of ORF1) and its flanking sequences, can provide efficient phylogenetic signal to reproduce genotyping results of the HEV full-length genome and may have the potential to serve as a consensus genomic region for future HEV genotyping, even when the synonymous sites among viral isolates are saturated. Moreover, it is worthy to note that the genomic region lengths are not limited to those used in this study and this study may also provide a useful tool based on calculating the evolutionary rate correlations to evaluate the phylogenetic performances of different genomic regions of different lengths that may be varied in a specific genotype analysis.

## Supporting Information

S1 Fig **Sliding-window analysis (window-size  = 500) of r-values between genomic regions and the full-length genome (data set 1).** The r-values were calculated for overlapping windows of 500 nucleotides shifted by one nucleotide and plotted against the midpoint of the window. The nucleotide positions represent the site positions in the alignment. The positions of the three open reading frames are shown along with the approximate positions within ORF1 of the methyl transferase (MeT), Ydomain (Y), papain-like cysteine protease (PCP), hypervariable region (HVR), X domain (X), helicase (Hel), and RNA-dependent RNA polymerase (RdRp).
(PDF)

S2 Fig **Phylogenetic analysis of HEV isolates based on a region with a low r-value.** Neighbor-joining trees were produced by using nucleotides of a region in ORF2 (5605–6004). Bootstraps values (>70) and scale bars are shown.
(TIF)
