# Supplementary figures and images for: Genome-Wide Comparisons of Phylogenetic Similarities between Partial Genomic Regions and the Full-Length Genome in Hepatitis E Virus Genotyping
Source: PLoS One. 2014 Dec 26;9(12):e115785. doi: 10.1371/journal.pone.0115785 (PMC4277416; doi:10.1371/journal.pone.0115785)

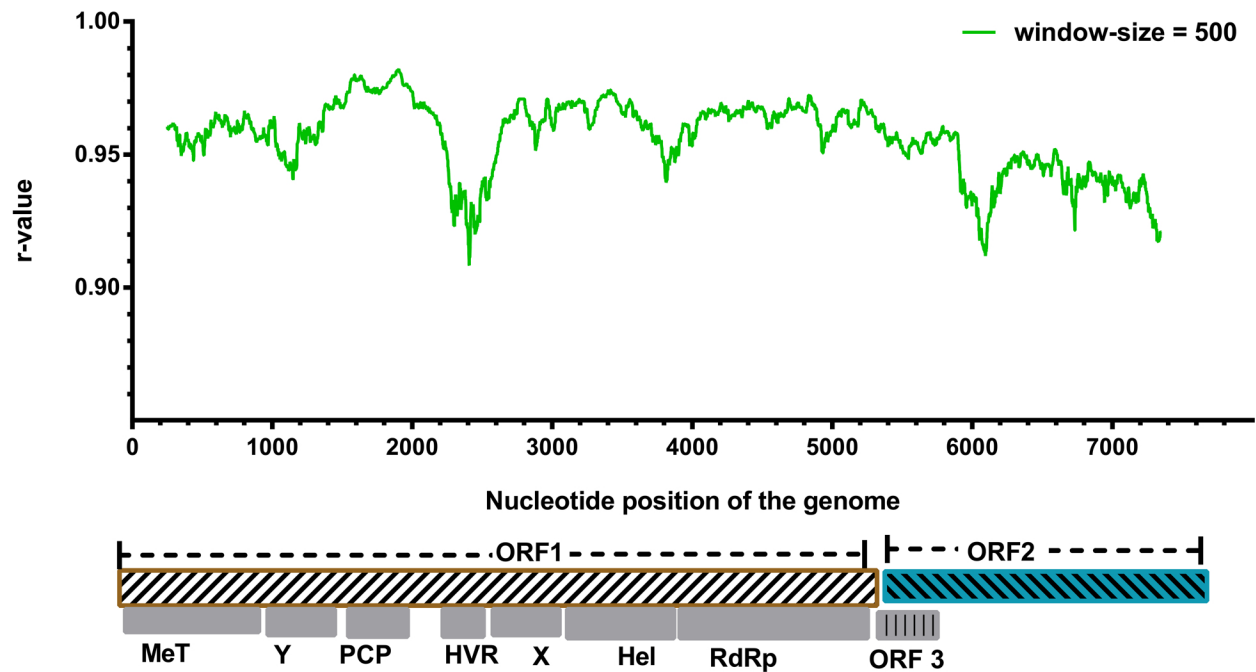

Supplement: S1 Fig — Sliding-window analysis (window-size = 500) of r-values between genomic regions and the full-length genome (data set 1). The r-values were calculated for overlapping windows of 500 nucleotides shifted by one nucleotide and plotted against the midpoint of the window. The nucleotide positions represent the site positions in the alignment. The positions of the three open reading frames are shown along with the approximate positions within ORF1 of the methyl transferase (MeT), Ydomain (Y), papain-like cysteine protease (PCP), hypervariable region (HVR), X domain (X), helicase (Hel), and RNA-dependent RNA polymerase (RdRp). (PDF) [file pone.0115785.s001.pdf]
